# Supplementary material for: Potentiation of Collagen Deposition by the Combination of Substance P with Transforming Growth Factor Beta in Rat Skin Fibroblasts
Source: Int J Mol Sci. 2024 Feb 3;25(3):1862. doi: 10.3390/ijms25031862 (PMC10855312; doi:10.3390/ijms25031862)
Supplement: Supplementary file 1 [file ijms-25-01862-s001.zip › Figure S1.pdf]

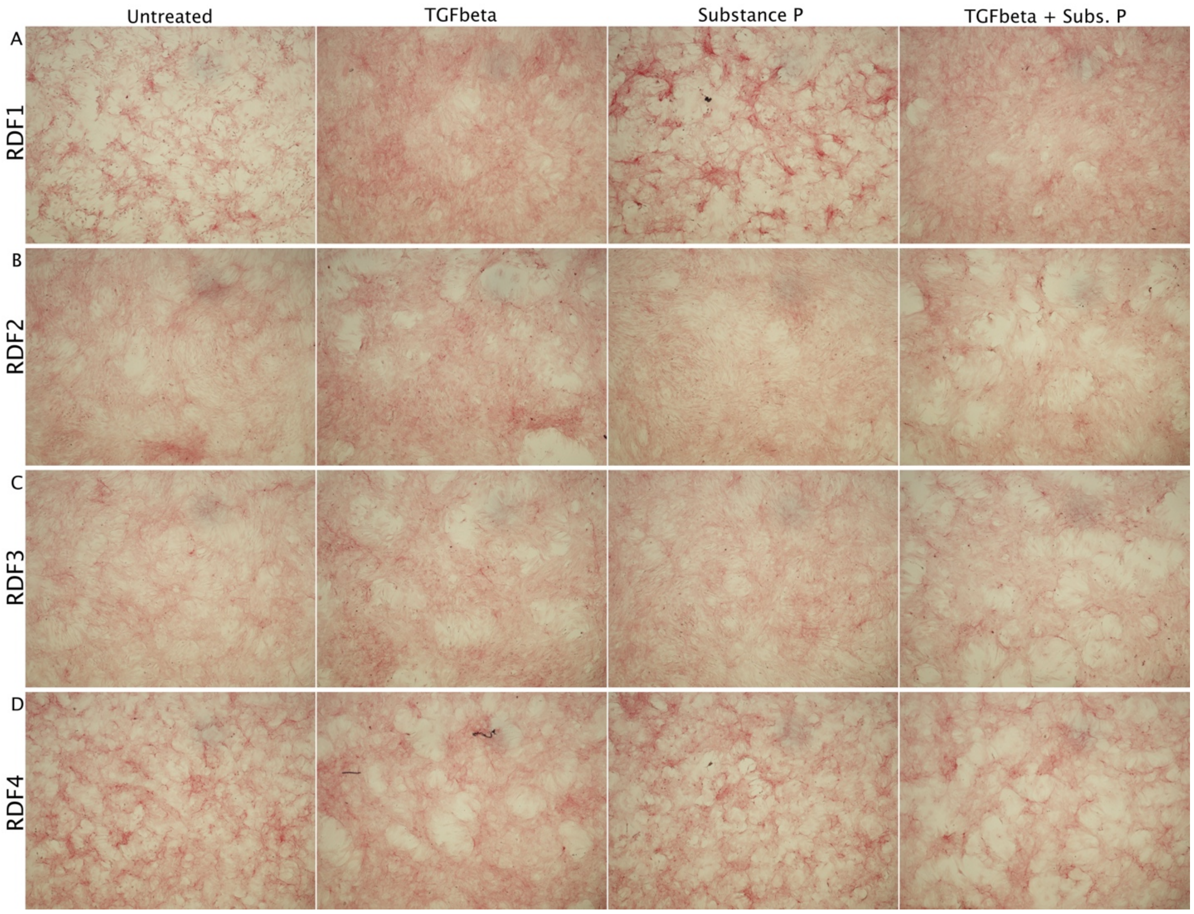


Figure S1. Sirius red staining of four dermal fibroblasts cell cultures from four different rats on day 5 of culture in medium containing 10% FCS. Each cell culture was seeded into 12 wells of a 48 well plate in 1% FCS. Cells were cultured for 3 days before the medium was removed and replaced with medium containing 10% FCS with and without TGFβ and or substance P 10% FCS. Cells were cultured for 5 days before fixing and staining with picrosirius red. (A) RDF1. (B) RDF2. (C) RDF3. (D) RDF4. Even collagen deposition across treatments and cell cultures is evident.
