# Supplementary material for: Potentiation of Collagen Deposition by the Combination of Substance P with Transforming Growth Factor Beta in Rat Skin Fibroblasts
Source: Int J Mol Sci. 2024 Feb 3;25(3):1862. doi: 10.3390/ijms25031862 (PMC10855312; doi:10.3390/ijms25031862)
Supplement: Supplementary file 1 [file ijms-25-01862-s001.zip › Table S1.pdf]

Table S1. Calculation of Potentiative Effect.

Day 5

	Untreated	TGFβ 5 ng/ml	Subs P 100 nM	TGFβ + Subs P Together	TGFβ + Subs P Separate	Potentiation Value
RDF1	0	0.0715	0.002	0.1185	0.0735	0.045
RDF2	0	0.09	0.019	0.2465	0.109	0.1375
RDF3	0	0.123	0.063	0.1175	0.186	-0.0685
RDF4	0	0.0485	-0.017	0.086	0.0315	0.0545

Day 12

	Untreated	TGFβ 5 ng/ml	Subs P 100 nM	TGFβ + Subs P Together	TGFβ + Subs P Separate	Potentiation Value
RDF1	0	0.069	0.0005	0.1135	0.0695	0.044
RDF2	0	0.0105	-0.048	0.058	-0.0375	0.0955
RDF3	0	0.0605	-0.0065	0.0975	0.054	0.0435
RDF4	0	0.077	0.012	0.1195	0.089	0.0305

The potentiative effect was calculated for each of four RDF cultures by firstly subtracting the mean untreated collagen matrix per cell values from each of the values obtained for each of the RDF treatments to ascertain the effect of the treatments compared to the untreated cells. The average for duplicate wells of 48 well plates for each treatment was then calculated and is shown. The total of the TGFβ and the substance P values were totaled and values for the four cultures is shown. Finally the potentiative value was calculated by subtracting this value from the value obtained for the TGFβ and substance P combination treatment. The results from day 5 and day 12 are shown.
